# The mitochondrial genome of *Murina huttoni rubella* (Chiroptera: Vespertilionidae) from China

**DOI:** 10.1080/23802359.2016.1180557

**Published:** 2016-07-08

**Authors:** Qiuping Zhang, Haiyan Cong, Wenhua Yu, Lingming Kong, Yingyong Wang, Yuchun Li, Yi Wu

**Affiliations:** a Key Laboratory of Conservation and Application in Biodiversity of South China, School of Life Sciences, Guangzhou University, Guangzhou, PR China;; b Marine College, Shandong University (Weihai), Weihai, Shandong, PR China;; c School of Life Sciences, Sun Yat-Sen University, Guangzhou, PR China

**Keywords:** Chiroptera, complete mitochondrial genome, Jiangxi; *Murina huttoni rubella*

## Abstract

*Murina huttoni rubella* is a common *Murina* species in China, with a medium forearm length and reddish brown hairs. In this study, based on a male *M. h. rubella* individual from Jiangxi, China, its complete mitochondrial genome was sequenced and analyzed. The genome is 16,707 bp in length, including 22 tRNA genes, two rRNA genes, 13 protein-coding genes and a control region. The composition and arrangement of genes are similar to other bats. Phylogenetic trees that covered all released complete mitochondrial genome of bats were constructed using Bayesian Inference and maximum likelihood methods. Both phylogenetic results showed that *M. h. rubella* and *M. ussuriensis* have closer phylogenetic relationship. The complete mtDNA genome sequence of *M. h. rubella* would provide valuable information for solving taxonomic and phylogenetic problem in future.

*Murina huttoni* is medium-sized *Murina* bat (Chiroptera: Vespertilionidae), with forearm length 32.8–35.4 mm (Bates & Harrison 1997). It is similar to *M. cyclotis* in pelage color, while it has longer and more slender ears. It distributes from northern Pakistan, northwest India to Southeast Asia (Corbet & Hill 1992). According to Wang (2003) and Smith and Xie (2008), populations distributed in China are often referred as *M. h. rubella* (type locality: Kuadun (Guadun), Fukien (Fujian)); while the Indian populations are often referred to the nominate race *M. h. huttoni* (Bates & Harrison 1997). Although variation of fur color has been observed between these two subspecies, such division lacks genetic evidence (Francis & Eger 2012). In this study, the mitochondrial genome of *M. h. rubella* was sequenced based on a male *M. h. rubella* that was collected from Jinggangshan National Natural Reserve in Jiangxi Province (114°11.647' E, 26°32.475' N, H = 819.3 m), China, which is about 350 km from type locality of *M. h. rubella*. The voucher specimen (GZHU 13478) was stored using 75% ethanol in Key laboratory of Conservation and Application in Biodiversity of South China, School of Life Sciences, Guangzhou University (Guangzhou). Total genomic DNA was isolated from muscle tissue using the traditional phenol-chloroform-isopentanol method. PCRs were carried out in 25 μl volume containing 12.5 μl Premix Taq (2 × EasyTaq PCR SuperMix (TransGen Biotech Co., Ltd, Beijing, China)), 0.4 μl each primer, 1 μl DNA and 10.7 μl ddH_2_O. The PCR program consisted of an initial pre-denaturation for 4 min at 94 °C, 36 cycles of 94 °C for 30 s and 50 ∼ 54 °C for 40 s and 72 °C for 1–3 min and a final extension step of 72 °C for 7 min. Aiming to amplify long sequence (>3 kb), long and accurate PCR (LA PCR) were also carried out, in 25 μl volume containing 0.25 μl LA-Taq, 2.5 μl 10 × LA PCR buffer, 4 μl dNTP (TAKARA Biotech Co., Ltd, Dalian, China), 0.4 μl each primer, 16.45 μl ddH_2_O and 1 μl template DNA. The LA-PCR program consisted of initial pre-denaturation for 1 min at 94 °C, 30 cycles of 98 °C for 8 s and 68 °Cfor 6 min, final extension step of 72 °C for 10 min.

The mitochondrial genome of *M. h. rubella* is a 16,707 bp circular molecule, containing 13 protein-coding genes, 22 tRNA genes, two rRNA genes and a control region. The sequence has been submitted in Genbank under the accession number KU521385. Among these 37 genes, most genes are encoded on the heavy (H) strand except one protein-coding gene (*ND6*) and eight tRNA (tRNA-Gln, Ala, Asn, Cys, Tyr, Ser, Glu and Pro), which encoded on the light (L) strand. Among the 13 protein-coding genes, *ATP8* and *ATP6* are overlapped by 43 nucleotides; *ND4L* and *ND4* are overlapped by seven nucleotides. These two reading-frame overlaps are both on the H strand. 13 protein-coding genes start with ATG, except for *ND2* (ATT), *ND3* (ATT) and *ND5* (ATA). And seven protein-coding genes stop with TAA, *Cyt b* ends with AGA. Five genes end with an incomplete stop codon TA- (*ND1*, *ND3*) and T– (*ND2*, *CO3*, *ND4*), which can be modified by the polyadenylation after transcript processing (Ojala et al. 1981). The length of the 22 tRNA genes range from 62 to 74 bp and can be folded into typical cloverleaf secondary structure, with an exception of tRNA-Ser. The control region is located between the tRNA-Pro and tRNA-Phe genes, and its length is 1259 bp, with high simple repeat sequence GCATAC.

In phylogenetic analysis, 47 released complete mitochondrial genome sequences of Chiropteran species from the GenBank were included and aligned using the MUSCLE (Edgar 2004). The alignment matrix was manually edited by Geneious Pro 4.8.2 (Newark, NJ). We used PartitionFinder 1.1.1 (Lanfear et al. 2012) to select the best partitioning scheme and best-fit models of nucleotide evolution. According to the Bayesian information criterion, the partitioning scheme with the best likelihood, separated the data sets among genes and among nucleotide positions in the different codons. In CIPRES Portal V3.3 (Miller et al. 2010), the phylogenetic relationship were inferred by the Maximum likelihood method (ML) using RAxML V8.2.4 (Stamatakis 2006, 2014) and Bayesian Inference (BI) using Mrbayes 3.2.2 (Ronquist et al. 2012). Both ML and BI received similar well-resolved phylogenetic trees. All *Murina* species were grouped together, and *M. h. rubella* and *M. ussuriensis* have closer relationship than *M. leucogaster* in the phylogenetic tree (Figure 1). Similar to other phylogenomic studies, our results also supported the assemblage of Yinpterochiroptera and Yangochiroptera (Teeling et al. 2005) (Figure 1). The sequence of *M. h. rubella* was the first complete mitochondrial genome sequence of *Murina* from China, which could provide valuable information for future taxonomic and phylogenetic studies.

**Figure 1.**
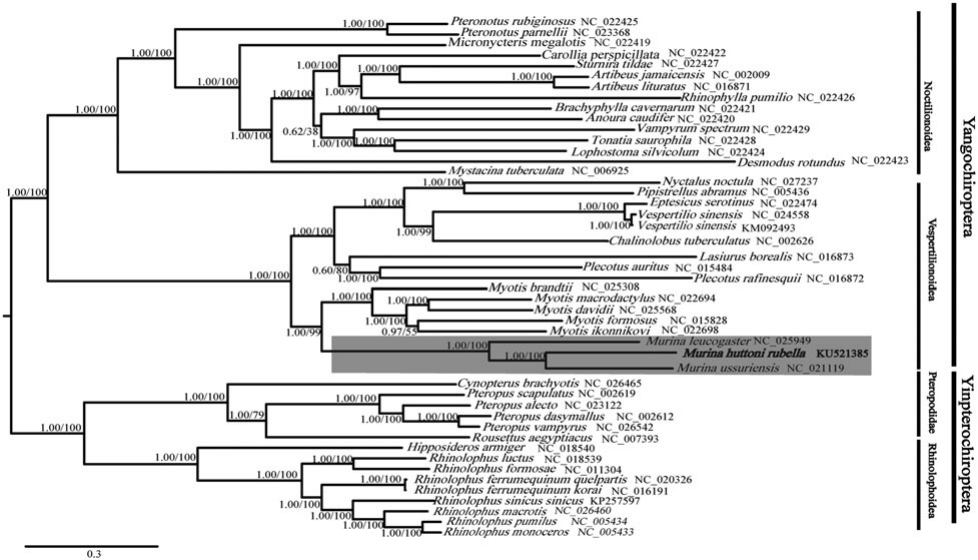
BI and ML phylogenetic trees of 47 species based on complete mitochondrial genome. Numbers above the nodes indicate posterior probabilities and bootstrap values, respectively. Branch length is based on ML trees, and the *Murina* clade is in shade.

## References

[CIT0001] Bates PJJ, Harrison DL 1997 Bats of the Indian Subcontinent. Sevenoaks: Harrison Zoological Museum.

[CIT0002] Corbet GB, Hill JE 1992 The mammals of the Indomalayan region: a systematic review. Oxford: Oxford University Press.

[CIT0003] Edgar RC 2004 MUSCLE: multiple sequence alignment with high accuracy and high throughput. Nucleic Acids Res. 32:1792–1797. 15034147 10.1093/nar/gkh340 PMC390337

[CIT0004] Francis CM, Eger JL 2012 A review of tube-nosed bats (*Murina*) from Laos with a description of two new species. Acta Chiropterologica. 14:15–38.

[CIT0005] Lanfear R, Calcott B, Ho SYW, Guindon S 2012 Partitionfinder: combined selection of partitioning schemes and substitution models for phylogenetic analyses. Mol Biol Evol. 29:1695–1701. 22319168 10.1093/molbev/mss020

[CIT0006] Miller MA, Pfeiffer W, Schwartz T 2010 Creating the CIPRES science gateway for inference of large phylogenetic trees. Proceedings of the Gateway Computing Environments Workshop (GCE). New Orleans, LA, 1–8.

[CIT0007] Ojala D, Montoya J, Attardi G 1981 tRNA punctuation model of RNA processing in human mitochondria. Nature. 290:470–474. 7219536 10.1038/290470a0

[CIT0008] Ronquist F, Teslenko M, Mark PVD, Ayres DL, Darling A, Höhna S, Larget B, Liu L, Suchard MA, Huelsenbeck JP 2012 MrBayes 3.2: efficient Bayesian phylogenetic inference and model choice across a large model space. Syst Biol. 61:539–542. 22357727 10.1093/sysbio/sys029 PMC3329765

[CIT0009] Smith AT, Xie Y 2008 A guide to the mammals of China. Princeton: Princeton University Press.

[CIT0010] Stamatakis A 2006 RAxML-VI-HPC: maximum likelihood-based phylogenetic analyses with thousands of taxa and mixed models. Bioinformatics. 22:2688–2690. 16928733 10.1093/bioinformatics/btl446

[CIT0011] Stamatakis A 2014 RAxML version 8: a tool for phylogenetic analysis and post-analysis of large phylogenies. Bioinformatics. 30:1312–1313. 24451623 10.1093/bioinformatics/btu033 PMC3998144

[CIT0012] Teeling EC, Springer MS, Ole M, Paul B, O'Brien SJ, Murphy WJ 2005 A molecular phylogeny for bats illuminates biogeography and the fossil record. Science. 307:580–584. 15681385 10.1126/science.1105113

[CIT0013] Wang YX 2003 A complete checklist of mammal species and subspecies in China: a taxonomic and geographic reference. Beijing: China Forestry Publishing House.

